# Reactivation of VX-Inhibited Human Acetylcholinesterase by Deprotonated Pralidoxime. A Complementary Quantum Mechanical Study

**DOI:** 10.3390/biom10020192

**Published:** 2020-01-27

**Authors:** Jorge Alberto Valle da Silva, Ander Francisco Pereira, Steven R. LaPlante, Kamil Kuca, Teodorico Castro Ramalho, Tanos Celmar Costa França

**Affiliations:** 1 Chemical, Biological, Radiological and Nuclear Defense Institute, Avenida das Americas, 28705, Guaratiba, 23020-470 Rio de Janeiro/RJ, Brazil; 2 Laboratory of Molecular Modeling, Chemistry Department, Federal University of Lavras, 37200-000 Lavras, MG, Brazil; ander.francisco@hotmail.com (A.F.P.); teo@dqi.ufla.br (T.C.R.); 3 INRS-Centre Armand-Frapier Santé Biotechnologie, 531 boulevard des Prairies, Laval, QC H7V 1B7, Canada; steven.laplante@iaf.inrs.ca (S.R.L.); tanosfranca@gmail.com (T.C.C.F.); 4 Department of Chemistry, Faculty of Science, University of Hradec Kralove, Rokitanskeho 62, 50003 Hradec Kralove, Czech Republic; 5 Laboratory of Molecular Modeling Applied to the Chemical and Biological Defense (LMCBD), Military Institute of Engineering, Praça General Tiburcio 80, Urca, 22290-270 Rio de Janeiro/RJ, Brazil

**Keywords:** acetylcholinesterase, VX, 2-PAM, QM/MM method

## Abstract

In the present work, we performed a complementary quantum mechanical (QM) study to describe the mechanism by which deprotonated pralidoxime (2-PAM) could reactivate human (*Homo sapiens sapiens*) acetylcholinesterase (*Hss*AChE) inhibited by the nerve agent VX. Such a reaction is proposed to occur in subsequent addition–elimination steps, starting with a nucleophile bimolecular substitution (S_N_2) mechanism through the formation of a trigonal bipyramidal transition state (TS). A near attack conformation (NAC), obtained in a former study using molecular mechanics (MM) calculations, was taken as a starting point for this project, where we described the possible formation of the TS. Together, this combined QM/MM study on AChE reactivation shows the feasibility of the reactivation occurring via attack of the deprotonated form of 2-PAM against the Ser203-VX adduct of *Hss*AChE.

## 1. Introduction

Studies on the high toxicity of insecticides and pesticides to mammals led to the development of organophosphorous (OP) nerve agents. These compounds have been synthesized and stockpiled as the most dangerous chemical warfare agents in the world since the 1930s [1,2,3,4,5,6,7,8]. The high toxicity of such agents comes from the irreversible inhibition of the enzyme acetylcholinesterase (AChE), a serine hydrolase that accomplishes cholinergic synapses throughout the nervous system through the hydrolysis of the neurotransmitter acetylcholine (ACh) [8,9,10,11]. The phosphorylation of the residue Ser203 in the catalytic anionic site (CAS) of human (*Homo sapiens sapiens*) AChE (*Hss*AChE) causes ACh accumulation in neuronal synapsis and neuromuscular junctions, which can lead to cholinergic syndrome, characterized by innerved-organ failures and ultimately death [11,12,13,14]. 

The reactivation of the OP-inhibited *Hss*AChE by pyridinium-aldoxime-based drugs has been the most relevant action for therapy against lethal OP poisoning [13,15,16] since 1956, when the 2-pyridinium methyl aldoxime (known as pralidoxime or 2-PAM) was first used in therapy against intoxication by parathion in Japan [17]. Today, the most accepted mechanism of action of such drugs [12,15], which are all 2-PAM derivatives [18], is the nucleophile attack on the Ser203-OP adduct, followed by the removal of the phosphate moiety from the enzyme’s active site [13,15,19]. However, there is no consensus yet about this mechanism due to the fact that none of the aldoximes developed so far are capable of reactivating AChE inhibited by all existing OPs [13,14,19]. Therefore, the full elucidation of the mechanism of this AChE reactivation reaction is crucial for the development of new and more efficient antidotes.

**Figure 1:**
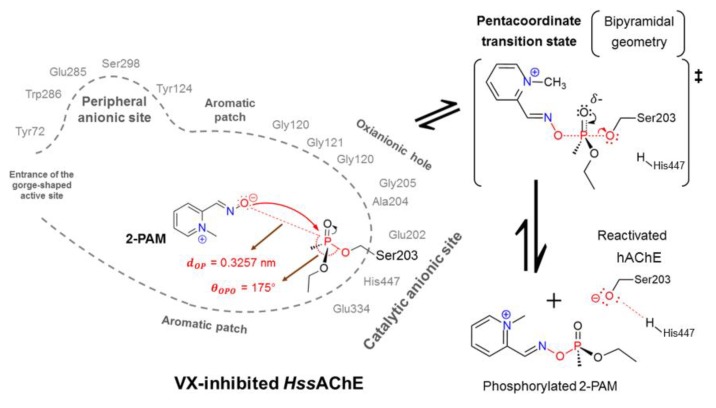
Representation of the near attack conformation (NAC) obtained before for deprotonated 2-PAM inside VX-inhibited *Hss*AChE after docking and molecular-dynamics (MD) simulations [20].

In our recent work, we presented molecular modeling studies meant to contribute to a better understanding of the reactivation mechanism of *Hss*AChE inhibited by the nerve agent O-ethyl S-(2-(diisopropylamino)ethyl) methylphosphonothioate (VX) [20]. In that case, classical molecular-dynamics (MD) simulations were performed, considering the near attack conformation (NAC) approach [20,21,22], on a model of *Hss*AChE based on the experimental structure of mouse AChE (PDB code 3ZLV). As a result, a NAC frame of deprotonated-2-PAM in the CAS of VX-inhibited *Hss*AChE was obtained [20]. The deprotonation of the aldoxime (−C=NOH) group may happen under physiologic conditions in the pathway towards the NAC [14,19,21,22,23], or before its entry into the AChE active site, according to the pKa = 7.68 of 2-PAM [24]. In our NAC, the distance between the O-atom of the aldoximate (−C=NO^−^) group and the P-atom of the phosphorylated Ser203 (*d**_OP_***) was 3.26 Å and the attack angle amongst the O- and P-atoms of the OP and the O-atom of Ser203 (*θ**_OPO_***) was 175° [20]. These values, according to the NAC concept early defined by [20,21,25], resemble the bonds to be formed and broken in a pentacoordinate transition state (TS) [26,27,28] of a bimolecular nucleophile substitution (S_N_2) mechanism (Figure 1) [29,30,31]. Experimental data for this reaction were published before by Nepovimova et al. [32]; however, data about its energetics of reactivation are still missing.

The combination of quantum mechanics (QM) and molecular mechanics (MM) techniques [33] is a valuable asset for the understanding of the enzyme reactivation mechanisms as well as to allow the modeling of larger systems, with a lower computational demand [34,35]. By performing such methodologies, Driant et al. [36] calculated the activation energy values for the reactivation reaction of VX-inhibited *Hss*AChE by protonated 2-PAM. The authors stated that such a mechanism was of the addition–elimination type after analyzing variations in lengths regarding *d_OP_ (O_Aldoximate_ – P_OP_)* and between the central P-atom of the OP and the O-atom of Ser203 (*P_OP_ – O_Ser203_*) [36]. In the present work, we took the NAC frame provided by MD simulation (Figure 1), reported before [20], to perform complementary studies regarding a comprehensive combined QM/MM methodology [36]. As a result, the theoretical efficiency of the deprotonated 2-PAM to reactivate the VX-inhibited *Hss*AChE was confirmed. Accordingly, the TS geometry was characterized and the reactivation energy barrier to achieve it was determined, similarly to what was previously described by Driant et al. [36].

## 2. Materials and Methods 

The structure of HssAChE inhibited by VX and complexed with 2-PAM used to perform the present work was the same model constructed for our former study [20]. This model was further verified to be identical to the experimental structures of HssAChE inhibited by VX available in the PDB.

**Figure 2:**
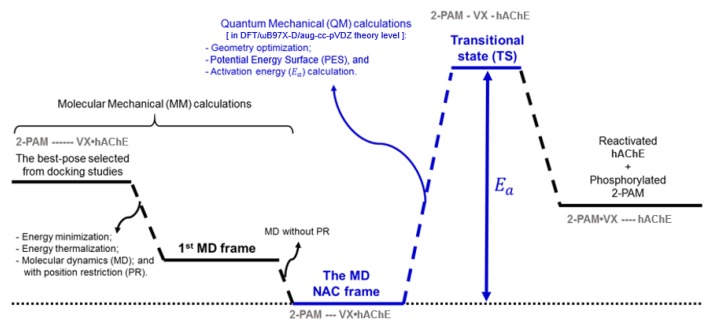
Energy profile obtained through the quantum mechanics (QM)/molecular mechanics (MM) methodology.

Figure 2 illustrates an energy profile suggested as a strategy to be employed to study the whole reactivation process by deprotonated 2-PAM through the S_N_2 mechanism [20,22], taking place within the narrow-gorge-shaped active center of VX-inhibited HssAChE, with the CAS at the bottom (Figure 1) [14,19]. The black lines on the left show the energy profile obtained through MM techniques performed in our previous works to figure out a refined NAC [20,21]. Such studies were needed to run the deeper theoretical studies through QM calculations of this work, represented by the blue lines (Figure 2). Initially, docking studies were carried out to compute the best pose, selected under the geometrical limitation stated by the NAC approach [20,21]. After this, the selected pose was submitted, as ligand, to 50 ns of MD simulation [20]. In order to elucidate the mechanism proposed in Figure 1, the NAC frame obtained from the MD simulation (Figure 1) was subsequently used as initial atomic coordinates (blue lines). The QM system consisted of such coordinates for the deprotonated 2-PAM, the residues of the catalytic triad (His447, Glu334 and the Ser203 complexed with nerve agent VX), amino-acid residues located in the oxyanion hole (Gly120, Gly121, Gly122, Ala204 and Gly205) and the residue Glu202, as illustrated in Figure 1. Then, from the NAC frame, a potential energy surface (PES) was constructed and the transition states were characterized by imaginary calculations. Thus, it was possible to determine the energy barrier involved in the reactivation of VX-inhibited AChE. All calculations were performed through the exchange–correlation functional ωB97X-D [37] in the framework of the density functional theory (DFT) [38], using the aug-cc-pVDZ basis set [39], by employing the Gaussian09 package [40], as indicated in Figure 2.

## 3. Results and Discussion

**Figure 3:**
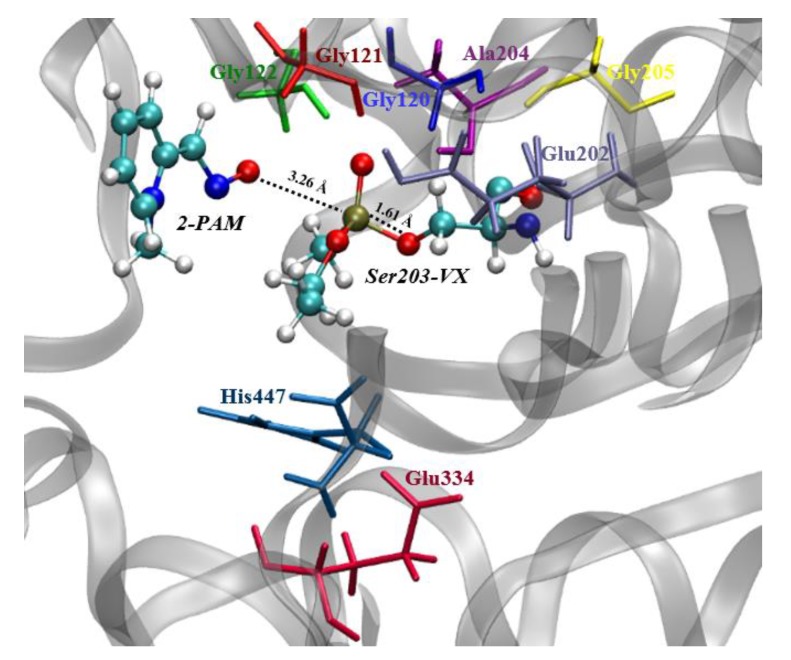
QM system (coloured atoms) after geometry optimization of the NAC frame.

Figure 3 shows the spatial arrangement of the QM system, obtained through the optimization calculation in DFT/ωB97X-D/aug-cc-pVDZ theory level from the atomic coordinates of the deprotonated 2-PAM at the NAC (Figure 1) obtained in our former MD calculations [20]. Accordingly, the O-atom of the −C=NO^−^ group was kept at a distance of 3.26 Å from the P-atom of the VX-Ser203 adduct, while at the *P_OP_ – O_Ser203_* bond, the length was 1.61 Å. In addition, the hydrogen atom of His447, which may stabilize the Ser203 after *Hss*AChE reactivation (Figure 1), was 7.84 Å away from the O-atom of Ser203 (Figure 3).

**Figure 4:**
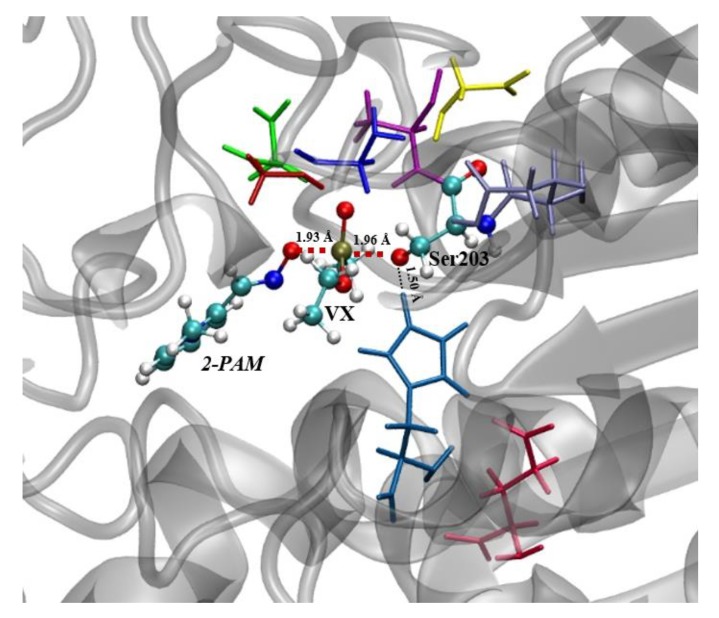
TS structure (QM system = coloured atoms) for the reactivation reaction of VX-inhibited *Hss*AChE by deprotonated 2-PAM.

When it comes to the deprotonated 2-PAM approximation towards the P-atom of the Ser203-VX adduct, a maximum-energy structure was achieved with the QM system geometry characterized by imaginary frequency calculations, as can be seen in Figure 4. As a result, such structure was built with the *O_Aldoximate_ – P_OP_* and *P_OP_ – O_Ser203_* binding lengths equal to 1.93 Å and 1.96 Å, respectively. As expected, both bond lengths (*O_Aldoximate_ – P_OP_* and *P_OP_ – O_Ser203_*) were such that they could hypothetically be formed and cleaved, pointing to a reactivation reaction through the S_N_2 mechanism. These findings suggest good agreement with the results from Driant et al. [36], who proposed that this reaction proceeds through an addition–elimination mechanism. In addition, the distance between one of the H atoms of His447 and the O-atom of Ser203 was finally 1.50 Å (Figure 4). This value shows that the His447 residue approaches considerably to stabilize Ser203 residue, which may support the formation of the products.

**Figure 5:**
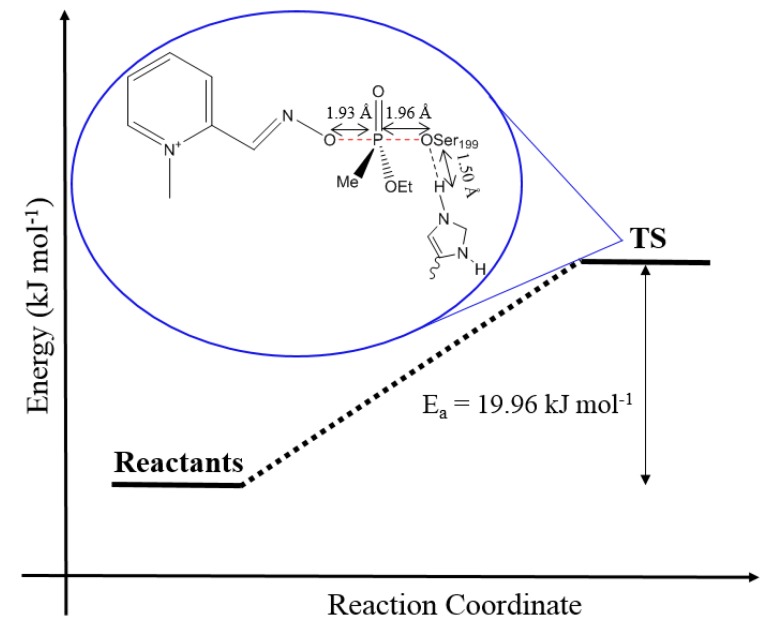
Activation energy profile for the formation of the complex 2-PAM/VX/Ser203.

The scheme in Figure 5 shows that the energy barrier between the reactants and the TS found for the reaction, multiplied by a correction factor, was of 19.96 kJ.mol^−1^. Such an energy value is lower than the energies reported by Driant et al. [at the QM (B3LYPD3/def2-SV(P) and QM/MM (CHARMM//B3LYP-D3/def2-TZVP (SP)) levels with 25.19 kJ mol^−1^ and 36.99 kJ mol^−1^, respectively] [36] for their best case of enzyme reactivation, which occurs with oxime in its deprotonated form, while residues His447 and Glu202 must be protonated for the reaction to occur. It is important to mention that Driant et al. adopted other functional and base sets (compared to those adopted in this work) to perform their calculations [36].

Therefore, the characterization of the possible TS built through a combined QM/MM methodology [41] showed the viability of the connections among reactants and TS, making it possible the proposition of the *Hss*AChE reactivation by deprotonated 2-PAM in an energetically favourable mechanism.

## 4. Conclusions

The results of classical MD simulations reported in our former study [20] were shown to be a good starting point for the QM calculations performed in this work, resulting in a comprehensive combined QM/MM approach to study the reactivation mechanism of OP-inhibited AChE. The NAC frame selected allowed for the assessment of a TS geometry compatible with an S_N_2 mechanism and the calculation of the activation energy profile. In this case, the deprotonated 2-PAM was stabilized inside the active-site gorge of VX-inhibited *Hss*AChE at a nucleophile attack position against the VX-Ser203 adduct. According to our results, the energy barrier for the AChE reactivation through deprotonated 2-PAM is lower than for the protonated 2-PAM. This corroborates our hypothesis that the reactivation mechanism might be triggered by the deprotonated form of the aldoxime instead of the protonated one. Also, analyzing the TS built, the reaction tends to form the products due to the approximation of His447 to the O-atom of Ser203. In addition, such deep-theoretical studies contribute to a better understanding of the mechanism of reactivation of *Hss*AChE by pyridinium-aldoximes. Furthermore, the selected theoretical approach showed similar results to those obtained by means of more robust and sophisticated methodologies, which required a higher computational cost. Therefore, such a methodology enables the study of more complex systems, which would, in theory, be much more computationally costly.
